# Endogenous overexpression of an active phosphorylated form of DNA polymerase β under oxidative stress in *Trypanosoma cruzi*

**DOI:** 10.1371/journal.pntd.0006220

**Published:** 2018-02-12

**Authors:** Diego A. Rojas, Fabiola Urbina, Sandra Moreira-Ramos, Christian Castillo, Ulrike Kemmerling, Michel Lapier, Juan Diego Maya, Aldo Solari, Edio Maldonado

**Affiliations:** 1 Microbiology and Micology Program, ICBM, Faculty of Medicine, University of Chile, Santiago, Chile; 2 Cellular and Molecular Biology Program, ICBM, Faculty of Medicine, University of Chile, Santiago, Chile; 3 Anatomy and Developmental Biology Program, ICBM, Faculty of Medicine, University of Chile, Santiago, Chile; 4 Molecular and Clinical Pharmacology Program, ICBM, Faculty of Medicine, University of Chile, Santiago, Chile; Bernhard Nocht Institute for Tropical Medicine, Hamburg, Germany, GERMANY

## Abstract

*Trypanosoma cruzi* is exposed during its life to exogenous and endogenous oxidative stress, leading to damage of several macromolecules such as DNA. There are many DNA repair pathways in the nucleus and mitochondria (kinetoplast), where specific protein complexes detect and eliminate damage to DNA. One group of these proteins is the DNA polymerases. In particular, Tc DNA polymerase β participates in kinetoplast DNA replication and repair. However, the mechanisms which control its expression under oxidative stress are still unknown. Here we describe the effect of oxidative stress on the expression and function of Tc DNA polymerase β To this end parasite cells (epimastigotes and trypomastigotes) were exposed to peroxide during short periods of time. Tc DNA polymerase β which was associated physically with kinetoplast DNA, showed increased protein levels in response to peroxide damage in both parasite forms analyzed. Two forms of DNA polymerase β were identified and overexpressed after peroxide treatment. One of them was phosphorylated and active in DNA synthesis after renaturation on polyacrylamide electrophoresis gel. This phosphorylated form showed 3-4-fold increase in both parasite forms. Our findings indicate that these increments in protein levels are not under transcriptional control because the level of Tc DNA polymerase β mRNA is maintained or slightly decreased during the exposure to oxidative stress. We propose a mechanism where a DNA repair pathway activates a cascade leading to the increment of expression and phosphorylation of Tc DNA polymerase β in response to oxidative damage, which is discussed in the context of what is known in other trypanosomes which lack transcriptional control.

## Author summary

Exposure of *Trypanosome cruzi* to oxidative stress leads to damage of several macromolecules such as DNA. DNA polymerases play a very important role in DNA repair after oxidative damage. One of them is Tc DNA polymerase β. In this work, two form of this DNA polymerase were identified and overexpressed in *T*. *cruzi* cells after hydrogen peroxide treatment been one of them a phosphorylated and highly active form. The increment of Tc DNA polymerase β was not correlated with changes in mRNA levels, indicating absence of transcriptional control. We propose a mechanism where hydrogen peroxide treatment activates a pathway leading to expression and phosphorylation of Tc DNA polymerase β in response to oxidative damage.

## Introduction

*Trypanosoma cruzi*, a flagellated protozoan, is the causative agent of Chagas´ disease (CD), which constitutes a major public health problem in Latin America. In endemic regions 10 million people are infected by the parasite and another 25 million are at risk of acquiring the infection [1]. There is currently no vaccine against *Trypanosoma cruzi* and the treatment of CD has limited efficacy and cause severe side effects [2]. Therefore there is a critical need to develop new chemotherapeutic agents and vaccines to control CD. Knowledge of the basic cellular processes of this parasite will help the development of new compounds to target specific processes of this parasite.

*T*. *cruzi* has a complex life cycle which includes different stages that alternate between invertebrate (insect vector) and vertebrate hosts, including humans. Epimastigote forms and infective metacyclic trypomastigotes occur in the insect vector and intracellular amastigotes and bloodstream trypomastigotes in the mammalian host [3]. Each stage of differentiation expresses a pattern of stage-specific proteins involved in the process of invasion and survival of the parasite.

The current chemotherapy for Chagas´ disease is based on benznidazole and nifurtimox, which are nitroheterocycle compounds; according to several studies they generate oxidative stress by *T*. *cruzi* nitroreductases [4, 5, 6]. Both type I nitroreductase in anaerobic and type II nitroreductase in aerobic conditions [5, 7], have been proposed for drug activation and promotion of DNA damage.

Reactive oxygen species (ROS) and reactive nitrogen species (RNS) derive from partly molecular oxygen and nitrogen reactants respectively, when produced in physiological quantities, play critical roles in the normal developmental process, and control signal transduction mechanisms that regulate cell proliferation, differentiation and death in higher eukaryotes [8, 9]. However, when ROS/RNS are produced in excess or for sustained periods they can rapidly oxidize proteins, lipids and DNA. ROS/RNS species and electrophilic metabolites can react with DNA, breaking bases and sugars, and cause nucleic acid chain cleavage [10]. However, several DNA repair mechanisms exist that restore DNA integrity, i.e. nucleotide excision repair (NER), base excision repair (BER) and a recombination system using the sister chromatid to repair DNA single and double stranded breaks [11, 12]. Most of the DNA repair processes need several DNA enzyme systems, but one of the last steps involves DNA polymerases that fill the gaps in the damaged DNA.

The susceptibility of trypanosomes to ROS/RNS in limiting *T*. *cruzi* replication and survival in infected cells and experimental animals has been reported [13]. The production of ROS/RNS is one of the most efficient mechanisms involved in the killing of *T*. *cruzi* bloodstream trypomastigotes by macrophages [14, 15, 16, 17] and also by cardiomyocytes *in vitro* [18], and to control *T*. *cruzi* infections *in vivo* [19]. Epimastigotes in the insect vector are exposed to ROS by the non-enzymatic Fenton reaction caused by Fe^+2^ of heme [20, 21].

*T*. *cruzi*, like other trypanosomatids, are exposed to several rapid changes since their life cycle occurs in different hosts exposed to various stresses, including oxidative stress. Unlike other eukaryotic organisms, trypanosomes lack the ability to control transcription [22]. However, potential components of signal transduction pathways have been described in trypanosomatids [23], which control translation and protein modification.

Different DNA polymerases have been described in *T*. *cruzi* which are related to DNA repair and oxidative stress: DNA polymerase β involved in the BER system and mitochondrial DNA replication, and the nuclear DNA polymerase *v* involved in the BER system. DNA polymerase *v*, DNA polymerase κ and DNA polymerase ζ participate in bypassing DNA lesions. The role of DNA polymerase β is poorly understood but it appears to be involved in DNA repair after oxidative stress, since its overexpression in transfected parasites after hydrogen peroxide treatment [24], and benznidazole oxidative stress increased parasite survival [25]. We have previously purified and characterized the native and recombinant DNA polymerase β from *T*. *cruzi* epimastigotes and generated polyclonal antibodies [26, 27]. To gain insights into the role of DNA polymerase β in *T*. *cruzi*, we have studied the effect of the oxidative stress induced by hydrogen peroxide on the expression and activity of DNA polymerase β in epimastigote and trypomastigote forms. Our results indicate that DNA polymerase β is overexpressed in those conditions. However, the mRNA levels of the enzyme do not change and the increasing levels seem to be at the post-translational level.

## Results

### Tc DNA polymerase β binds to the kinetoplast DNA

**Fig 1:**
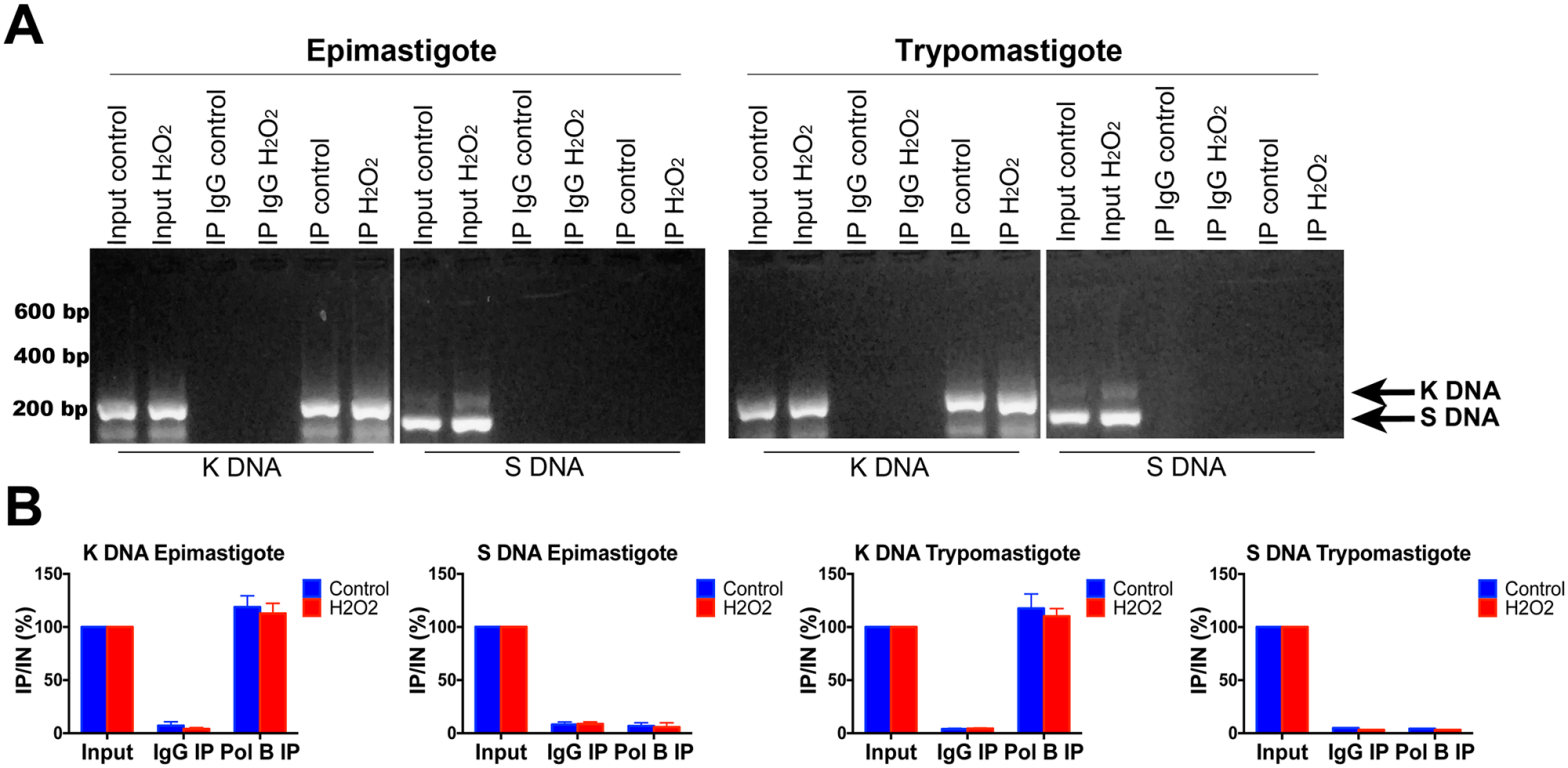
Crosslinking of Tc DNA polymerase β to kinetoplastid DNA. (A) *T*. *cruzi* epimastigote and trypomastigote forms were treated with peroxide. Proteins were crosslinked to the DNA with formaldehyde and then DNA was sheared and immunoprecipitated with antibodies against Tc DNA polymerase β (αDNA β-Pol) or control IgG. Revert-crosslinked DNA precipitated (IP) was purified and amplified by PCR to amplify nuclear satellite DNA (S DNA) or kinetoplastid DNA (K DNA). (B) Quantification of PCRs amplifying S DNA and K DNA are indicated above the gel panel. Results are indicated as the mean +/- SD of three independent experiments. Input (IN) corresponded to 2% of total starting DNA and precipitated DNA (IP) represents 50%. * indicates *p* < 0.05.

Tc DNA beta polymerase has been immunolocalized inside the mitochondria of *T*. *cruzi* organisms. However, there are no supporting studies indicating a physical interaction between Tc DNA polymerase β and kinetoplast DNA and whether or not this interaction is affected by H_2_O_2_ stress. To investigate this interaction, immunoprecipitated DNA from epimastigote and trypomastigote cultures treated or not with H_2_O_2_ were amplified by end point PCR with specific primers either for the kinetoplast or nuclear DNA (Table 1). As can be seen in Fig 1, either from H2O2-treated and from untreated cells, kinetoplast DNA was successfully precipitated by Tc DNA β polymerase antibody, since positive PCR amplification was observed. However, no positive amplification was observed with nuclear DNA, indicating a negative interaction with Tc DNA polymerase β antibody. These results suggest that there is a direct interaction between Tc DNA polymerase β and kinetoplast DNA in epimastigote and trypomastigote developmental forms, which is in agreement with the mitochondrial localization described elsewhere [28].

**Table 1:** Oligonucleotides used in this paper.

Name	Sequence (5’– 3’)	Utilization
***TCBF1***	CTCACGTTTACCGGCAGCAA	*T*. *cruzi* DNA polymerase β amplification (qPCR and Northern blot).
***TCBR1***	AACGTCTCCATCTTCGCCGA	*T*. *cruzi* DNA polymerase β amplification (qPCR and Northern blot).
***TCTF1***	GCTTTGAGCTTTGATGGCT	*T*. *cruzi* actin (qPCR and Northern blot).
***TCTR1***	AAAAAAACGCGATATAAA	*T*. *cruzi* actin (qPCR and Northern blot).
***TCZ1***	CGAGCTCTTGCCCACACGGGTGCT	*T*. *cruzi* satellite DNA amplification
***TCZ2***	CCTCCAAGCAGCGGATAGTTCAGG	*T*. *cruzi* satellite DNA amplification
***#121***	AAATAATGTACGGG(T/G)GAGATGCATGA	*T*. *cruzi* kinetoplast DNA amplification
***#122***	GGTTCGATTGGGGTTGGTGTAATATA	*T*. *cruzi* kinetoplast DNA amplification

### Tc DNA polymerase β protein levels increase in response to H_2_O_2_ damage

**Fig 2:**
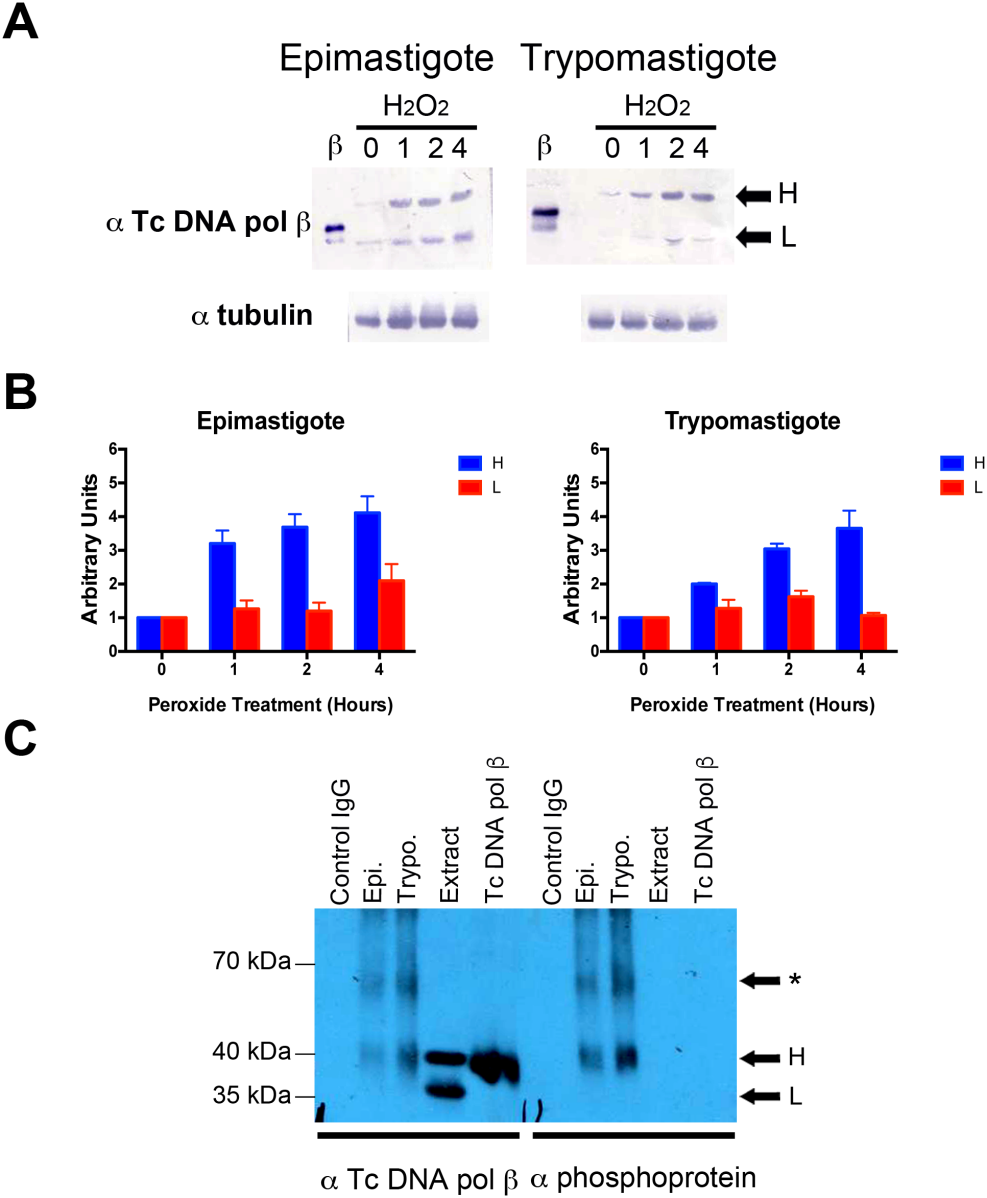
Effect of H_2_O_2_ treatment on the expression of Tc DNA polymerase β. (A) Western blot analysis of epimastigotes and trypomastigotes treated with H_2_O_2_ for 0, 1, 2 and 4 hours as indicated at the top of the figure. 5x10^5^ epimastigotes and 1x10^6^ trypomastigotes were lysed and loaded in 8,5% SDS-PAGE gels. Tc DNA polymerase β was detected using a specific antibody (α Tc Pol β). H and L indicate the large and the small forms of the enzyme, respectively. β indicates the presence of recombinant Tc DNA polymerase β. Detection of α tubulin was added as loading control and is shown at the bottom of the figure. (B) Quantification of results of panel (A). The detected bands corresponding to H and L were quantified and compared to the loading control (α tubulin). Results are expressed in arbitrary units and represent the mean +/- SD of three independent experiments. (C) Western blot analysis of Tc DNA polymerase β immunoprecipitated from peroxide-treated epimastigote (Epi.) and trypomastigote (Trypo.) cells. Cell extracts were incubated with antibodies against Tc DNA polymerase β or IgG crosslinked to protein-A agarose beads (see Methods). Precipitated material was loaded onto 8,5% SDS-PAGE gels and transferred to PVDF membranes and probed with antibodies against Tc DNA polymerase β or phosphoprotein. In the figure, extract indicates proteins before immunopurification and Tc DNA pol β indicates the recombinant enzyme. H and L indicate the presence of the large and small forms of Tc DNA polymerase β. * indicate the presence of trimers o tetramers formed upon TCA precipitation.

To investigate the effect of oxidative stress in the DNA repair function of DNA polymerase β, we treated both epimastigote and trypomastigote cells with H_2_O_2_ for different periods of time and proteins were analyzed by western blot. As can be seen in Fig 2A and 2B, protein levels of Tc polymerase β were increased in both epimastigote and trypomastigote forms and the increment was dependent on the treatment time. The high (H) and low (L) molecular weight protein forms identified were quantified and the results are indicated in Fig 2B. In epimastigote and trypomastigote forms the H-form showed an increment of >4-fold compared to the control (0 hours treatment). However, the L-form showed an increment of >4-fold after 1 hour of peroxide treatment in epimastigotes, whereas in trypomastigotes a 2-fold increment was observed after 2 hours of peroxide treatment. A control protein, tubulin, did not show increase upon treatment either in trypomastigote or epimastigote forms, indicating that there is a selective increase of the Tc DNA polymerase β enzyme after treatment with hydrogen peroxide and not a global change in protein synthesis. To evaluate post-translational modification that explains the presence of two protein forms in epimastigote and trypomastigote cells, we first evaluated the phosphorylation state of Tc DNA polymerase β in protein extracts. For this we prepared protein extracts from epimastigotes and trypomastigotes and Tc DNA polymerase β was immunopurified from these extracts. The resulting material was subjected to western blot analysis. In the precipitated material, the slower migrating polypeptide (H) is phosphorylated in trypomastigote cells, since it can react with antibodies against phosphoaminoacids (Fig 2C). These observations agree with previous results in epimastigotes [27]. We did not detect a change in the mobility of the slower migrating band upon treatment with N- or O- glycosidases, suggesting the enzyme does not contain sugar moieties.

### The slower migrating form of Tc DNA polymerase β is active in DNA synthesis

**Fig 3:**
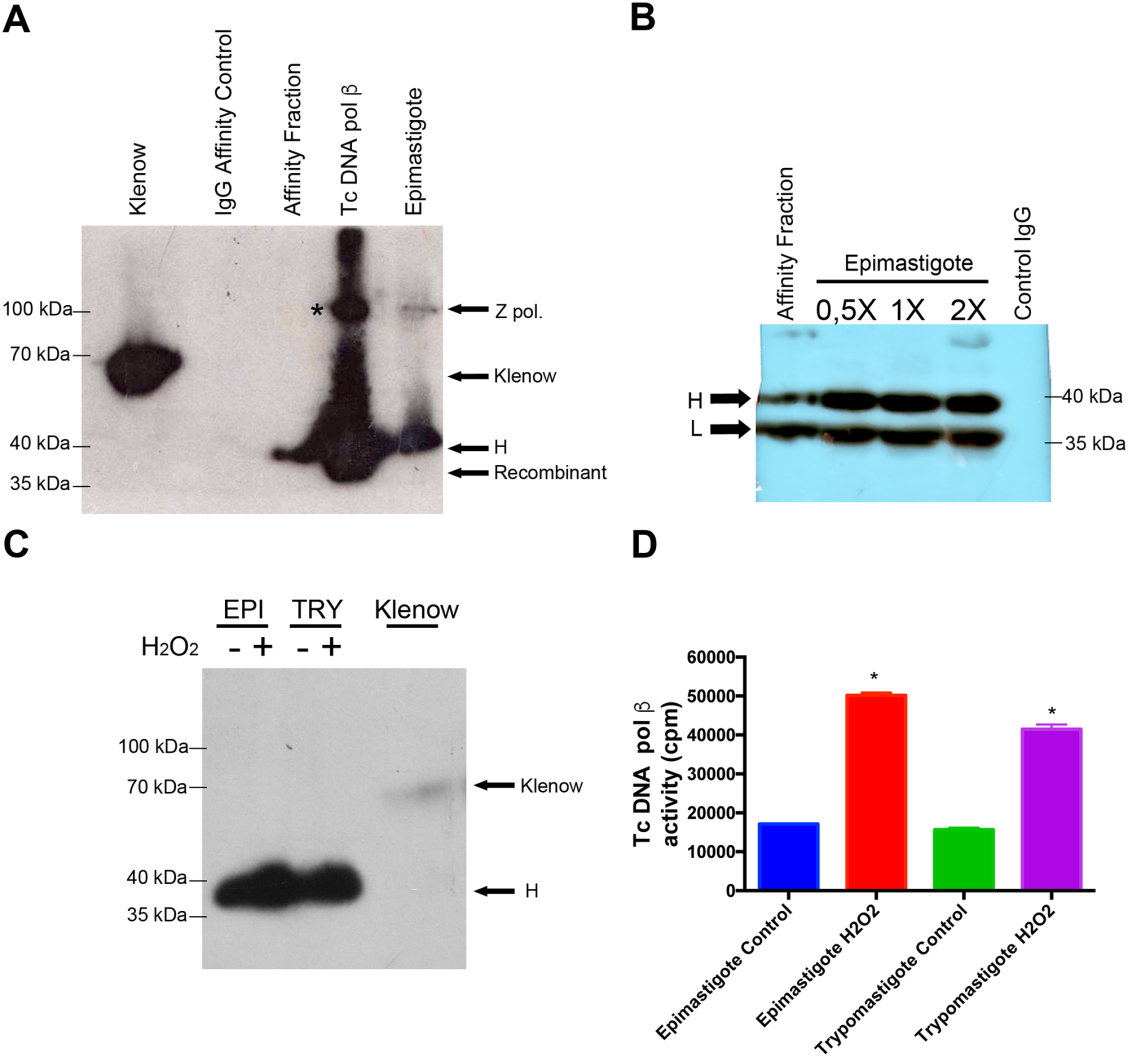
Measurement of DNA synthesis in activity gels. (A) SDS-PAGE activity gels were loaded with 100 pg of Klenow fragment (control), 15 μl of purified Tc DNA polymerase β (Affinity Fraction), 500 pg of recombinant Tc DNA polymerase β and 1 μg of epimastigote cell extract (Epimastigote). Then the gel was processed to evaluate in-gel DNA synthesis (see Methods). Z pol indicates the position of Tc DNA polymerase ζ detected in the epimastigote fraction. H indicates the position of the large form of Tc DNA polymerase β (detected in affinity fraction and epimastigote). (B) Western blot of the samples analyzed in (A). Three different amounts of epimastigote cell extract were evaluated: 0.5, 1 and 2 μg (0.5X, 1X and 2X respectively). H and L indicate the migration of large and small forms of Tc DNA polymerase β. (C) SDS-PAGE activity gel was loaded with 1 μg of epimastigote (EPI) and trypomastigote (TRY) cell extracts either from peroxide-treated organisms (+) or without treatment (-). (D) Radioactive protein bands were excised from the gel and quantified by liquid scintillation. Results are expressed in cpm (counts per minute) and represent the mean +/- SD of four independent experiments. * indicates *p* < 0,05 between non-treated v/s peroxide-treated organisms.

Earlier observations indicated that the native Tc DNA polymerase β could be purified as a doublet and only the slower migrating form is active in DNA synthesis [26]. To investigate which form of the enzyme is the active one, we purified Tc DNA polymerase β from crude cell extracts using affinity chromatography with antibodies covalently attached to protein A-agarose. The extracts were incubated with the resin, washed and the bound enzyme was eluted with a buffer containing SDS and urea. The eluted fractions were analyzed through SDS-PAGE and polymerase activity was evaluated in an activity gel. As can be seen in Fig 3, only one polypeptide from the purified fraction is active and co-migrates with the high molecular weight band of Tc DNA polymerase β (H). In crude epimastigote extracts there is a main active polypeptide, which migrates along with the active polypeptide from the affinity-purified fraction, and a weak active polypeptide, which is larger than Tc DNA polymerase β. This protein band might correspond to the Tc DNA polymerase ζ catalytic subunit (Accession EKF26846.1), which is composed of 980 amino acids and has a calculated MW of 108 kDa. The recombinant Tc DNA polymerase β is also active in this assay and migrates slightly below the native enzyme as it is detected in western blot analysis. The presence of only one active polypeptide in the affinity-purified fractions is not because only one polypeptide was retained by the antibodies, since a western blot analysis revealed that both polypeptides are present in both affinity purified and crude extracts (Fig 3B). Also, the activity of the 45 KDa polypeptide increased in both epimastigote and trypomastigote developmental forms treated with H_2_O_2_ (Fig 3C and 3D).

### mRNA levels of Tc DNA polymerase β do not increase in response to H_2_O_2_ treatment

**Fig 4:**
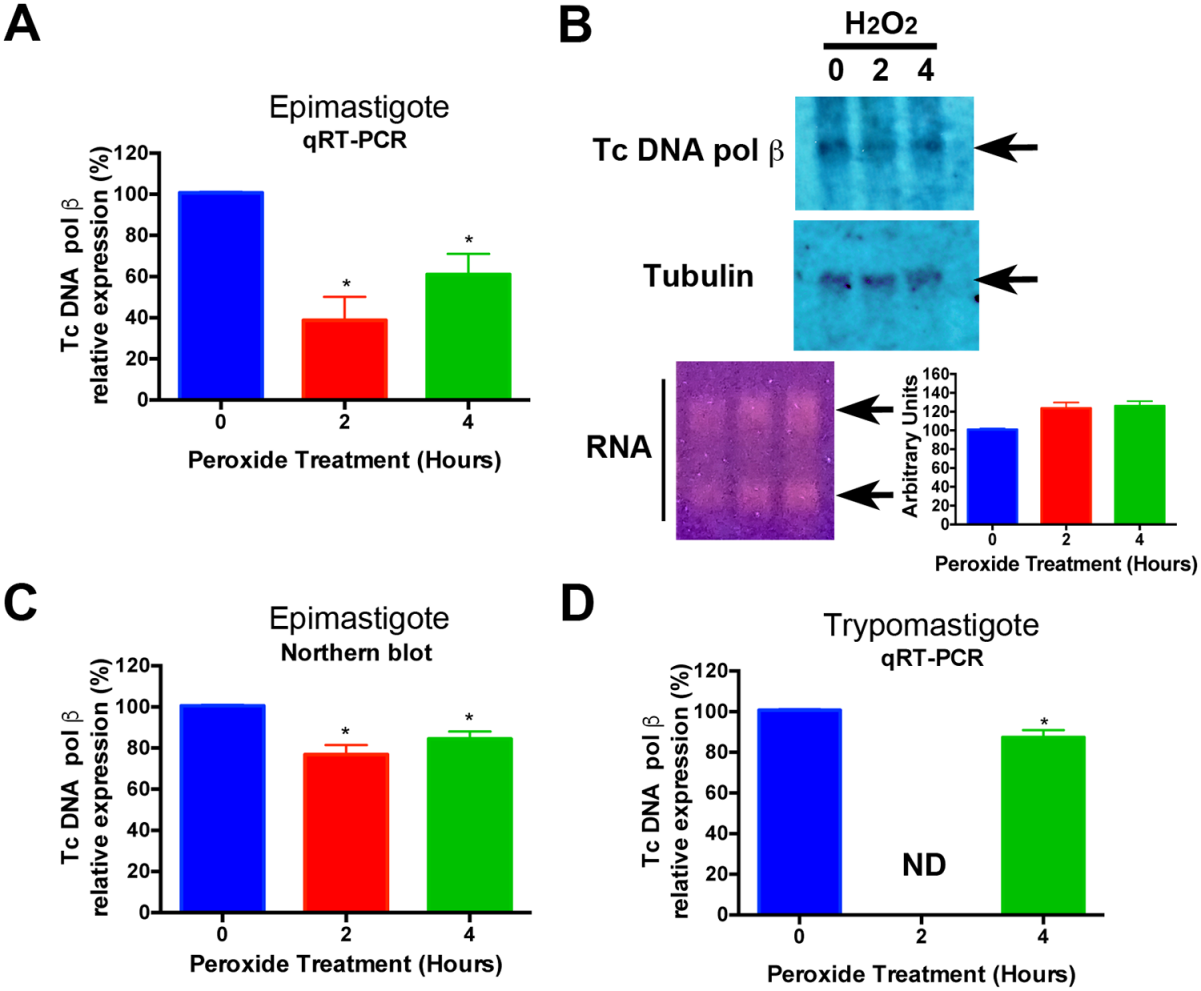
Expression of Tc DNA polymerase β in H_2_O_2_-treated cells. (A) Levels of mRNA were detected by qRT-PCR. cDNA samples were synthesized using total RNA extracted from epimastigote cells treated with H_2_O_2_ for 0, 2 and 4 hours. Results are presented as mean +/- SD of three independent experiments. Experiments were normalized using actin as an internal control. * indicates *p* < 0.05. (B) Levels of Tc DNA polymerase β mRNA were analyzed by Northern blot. Arrows indicate the position of Tc DNA polymerase β mRNA. Actin mRNA was detected as an internal control. Bottom panel shows the integrity of the total RNA analyzed by Northern blot (where arrows indicate the position of ribosomal RNA) and its quantification. (C) Quantification of three independent Northern blot analyses. Results are expressed as mean +/- SD. * indicates *p* < 0.05. (D) The levels of mRNA of Tc DNA polymerase β were detected by RT-qPCR. cDNA samples were synthesized using total RNA extracted from trypomastigote cells treated with H_2_O_2_ for 0 and 4 hours. Results are presented as mean +/- SD of three independent experiments. Experiments were normalized using actin as an internal control. * indicates *p* < 0.05.

To investigate the levels of Tc DNA polymerase β mRNA in H_2_O_2_-treated epimastigotes, we isolated RNA from treated cells and it was reverse transcribed and quantified by qRT-PCR. The levels of Tc DNA polymerase β mRNA from epimastigotes cells at 0, 2 and 4 hours of treatment with H_2_O_2_ are shown in Fig 4A. It can be seen that the levels of mRNA for the enzyme tend to decrease upon treatment with hydrogen peroxide (60% after 2 hours of treatment and 40% after 4 hours). However, we do not know whether or not there is a change in the total mRNA or if it is the result of peroxide damage to the mRNA and this is not able to serve as a template for the reverse transcriptase. To test the effect of peroxide treatment on mRNA integrity we evaluated Tc DNA polymerase β levels by Northern blot. Fig 4B and 4C show that mRNA levels decreased nearly 20% after 2 hours of treatment and 10% after 4 hours. These results correlate with results from RT-qPCR, although the differences were greater than the Northern blot results. In addition, the levels of Tc DNA polymerase β mRNA from trypomastigote cells at 0 and 4 hours of treatment with H_2_O_2_ were also measured by qRT-PCR (Fig 4D). It can be seen that the mRNA levels for the enzyme decrease near 15% after 4 hours of peroxide treatment, unfortunately the amount of total RNA obtained in those experiments was not enough to analyze Tc DNA polymerase β mRNA levels by Northern blot.

Altogether, these results indicate that there is not a transcriptional control of the expression of the Tc DNA polymerase β gene since the mRNA levels did not increase considerably.

## Discussion

Eukaryotic cells contain multiple DNA repair pathways that maintain genomic DNA integrity. The Base Excision Repair (BER) pathway protects DNA by removing oxidized, methylated or deaminated bases by the action of glycosylases [29]. These DNA sites are called apurinic/apyrimidinic (AP) sites and arise mainly as base oxidation from reactive oxygen species (ROS), among others. The BER pathway is distinguished from other DNA repair pathways by the removal of the base lesion. Several steps are necessary to accomplish DNA repair; one of the final steps involves DNA synthesis to fill the gap to ligate DNA strands. In mammalian systems DNA polymerase β contributes to filling the gap, removing the 5´deoxiribose phosphate (dRP) termini by a dRP lyase activity to later make single-nucleotide gap-filling DNA synthesis. DNA polymerase β belongs to the X family of DNA polymerases and is found in all vertebrate species as a 39-kDa protein lacking proofreading 3´-or 5´-exonuclease activity but containing 5´-dRP lyase and AP lyase activities [30]. A homologous DNA pol β gene has been found in yeast [31, 32]. Lower eukaryotic organisms such as Trypanosomatids also possess a DNA polymerase β and it might be involved in DNA repair. DNA pol β is considered the simplest naturally occurring DNA polymerase, making it an ideal model for studies to develop substrates/inhibitors that could specifically inhibit it.

In this study we examined the effect of oxidative stress on *T*. *cruzi* (Tc) DNA polymerase β expression, showing the increment of protein levels of a phosphorylated form of this DNA polymerase during H_2_O_2_ treatment, although mRNA levels evaluated by qRT-PCR and Northern blot did not show significant differences. The oxidative stress effect in *T*. *cruzi* has been documented *in vitro* [13, 33] and *in vivo* [19]. It is estimated that BER is the main DNA repair pathway acting upon ROS [34] and it has been described that this pathway is active in nuclei and mitochondria under oxidative stress [33]. In addition, transfected parasites overexpressing Tc DNA pol β showed increased survival after treatment with benznidazole (BZ), the classic anti-Chagas drug and a potent oxidative stressor, compared to non-treated cells [25]. These results also suggest that Tc DNA pol β exerts the DNA repair function on kDNA rather than on the nuclear DNA as previously suggested [24] and according to our results. Other DNA polymerases associated with DNA repair can to synthetize DNA bypassing DNA lesions, i.e. DNA polymerase ζ, DNA polymerase η and DNA polymerase κ. DNA polymerase ζ has not been characterized in parasites yet. However, overexpression of polymerases η and κ in transfected parasites displayed resistance to benznidazole and hydrogen peroxide in the same way as polymerase β [25, 35].

Oxidative stress with hydrogen peroxide diminishes cell viability of *T*. *cruzi* epimastigotes and trypomastigotes [33]. *T*. *cruzi* DNA damage and repair of epimastigotes in the presence of Nifurtimox or BZ induces nuclear DNA and kDNA damage but the effect is reversed when the drugs are removed, and the parasites are incubated in fresh medium [36]. The results presented in this work suggest the presence of a robust DNA repair machinery in *T*. *cruzi*.

Major mechanisms of gene regulation in *T*.*cruzi* and other kinetoplastids appear to be post-transcriptional since transcriptional units are polycistronic and promoters do not have regulatory sequences as in higher eukaryotic organisms [37], therefore signaling pathways regulating gene expression might target molecules regulating RNA processing/turnover and protein modification. Our results showed the presence of an active phosphorylated form of Tc DNA polymerase β, which correlates with a high molecular weight active form of this DNA polymerase whose levels are increased when parasite cells are exposed to peroxide. The protein levels of this DNA polymerase form are increased during peroxide treatment in the epimastigote and trypomastigote forms despite mRNA levels tend to decrease, suggesting a post-transcriptional regulation and a possible phosphorylation-mediated pathway dependent on oxidative stress. Interestingly, only the high molecular weight form of the polymerase has DNA synthesis activity suggesting that phosphorylation assist to the proper refolding of the protein.

The identification of the pathway associated with oxidative stress found in our experimental approach is part of the projections of this work. One of the putative pathways that could be related is MAP kinase pathways, which have been identified regulating cell growth, apoptosis and stress responses in higher eukaryotics [38]. In yeast and other fungus-related organisms, a conserved pathway has been described called the SPAK-pathway (stress protein activated kinases), which involves MAPK proteins and AP-1-like transcription factor [39]. In the case of trypanosomatids, they must respond to extracellular and intracellular signals as they adapt rapidly to new environments within their varied hosts. However, the molecular mechanisms of parasite responses to environmental changes are still unknown.

Recent studies in trypanosomatids have described stress-response mechanisms involving post-transcriptional regulation. For example, rapid shifts to low temperatures in the presence of cis-aconitate allow differentiation of bloodstream to procyclic forms of *Trypanosoma brucei* [40]. A protein phosphatase PIP39 and a potential RNA-binding protein are involved in this process [41, 42]. Studies in the same parasite from procyclic differentiation to mammalian infective forms after heat shock show changes in the compartmentalization of mRNA, predominantly untranslated and associated with proteins from the cytosolic fraction to polysomal fraction after 1 hour of heat shock treatment [43]. Several proteins such as the zinc-finger ZC3H11, polyA binding proteins, helicase, aggregation-prone protein and a 5–3 exonuclease are involved in this mechanism of mRNA storage and degradation of non-translated mRNAs. These factors are RNA-binding proteins (RBPs) and are the hallmark of mRNA stabilization, affecting the whole gene expression process in Trypanosomatids [44]. ZC3H11 binds to the 3’-UTRs of chaperone mRNAs and is required both for target mRNA retention and for cell survival after heat shock [45]. This zinc finger protein also binds to MKT1, which is a protein associated with stress resistance, and two polyA binding proteins required for translation initiation [46]. Several reports have shown that ZC3H11 is phosphorylated and the most likely protein kinase is casein kinase 1 [47].

Finally, according to our results a phosphorylated form of Tc DNA polymerase β is present in epimastigote and trypomastigote cells. This protein form is associated with a slower migrating band detected by SDS-PAGE and western blot. Overexpression of the phosphorylated form of Tc DNA polymerase β is observed in both epimastigote and trypomastigote cells, indicating a putative stress response that could lead to stress-related pathway activation where protein kinase induction could be involved. This induction would lead to two putative responses: 1. function modulation of Tc DNA polymerase β by direct phosphorylation; and 2. changes in mRNA stabilization by phosphorylation of mRNA chaperones associated with 3’ or 5’ UTR, leading to high/low translation rate. However, further studies must be performed to identify the exact mechanism involved in phosphorylation of Tc DNA polymerase β and what are the stress-related pathways activated during this process and to evaluate the mechanisms recently described involving post-transcriptional mRNA stabilization and protein kinases such as casein kinase 1.

## Methods

### Epimastigote and trypomastigote extract preparation

Epimastigote cells (6 x 10^8^) were harvested and washed with PBS. Cells were then resuspended in 5 ml of lysis buffer (50 mM Tris-HCl (pH 8.0), 1 mM EDTA, 1 mM EGTA, 500 mM KCl, 5 mM DTT, 0.5% v/v NP-40, 0.1 v/v Triton X-100, 10% v/v glycerol, 0.5 mM PMSF, 10 mM TSK) supplemented with a tablet of protease inhibitor and Phospho-Stop (Roche, Germany). Lysed cells were mildly sonicated at 4°C and centrifuged at 15000 rpm. Supernatants were collected and dialyzed against the lysis buffer without NP-40 or Triton X-100 but containing 50 mM KCl. Usually the extract contained 5 mg/ml of total protein as measured by the method of Bradford [48]. The extract was stored at -80°C until used to purify Tc DNA β polymerase by affinity chromatography. Cell extract from hydrogen peroxide-treated trypomastigotes (50 x 10^6^ cells) was done exactly as described before, except that the cells were resuspended in 500 μl of buffer. Extracts were dialyzed and stored to -80°C until use.

### Trypomastigote cell culture and H_2_O_2_ treatment

Green Monkey (*Cercopithecus aethiops*) renal fibroblast-like cells (VERO cells, ATCC CCL-81, USA) were grown in RPMI medium enriched with 5% fetal bovine serum (FBS) and antibiotics (penicillin-streptomycin). Cells were grown at 37°C in a humid atmosphere at 5% CO_2_ for 96 hours, replacing the medium every 24 hours. After confluence, VERO cells were incubated with a culture of epimastigotes in late stationary phase (strain Y), which increases the percentage of trypomastigotes to approximately 5%. Trypomastigotes then invaded fibroblasts and replicated intracellularly as amastigotes. After 72 hours, amastigotes transform back to trypomastigotes that lyse host cells. Parasites were recovered by low-speed centrifugation (500 x g), thus obtaining trypomastigotes in the supernatant and amastigotes in the sediment. The cells were suspended at 5 x 10^5^ cells/ml in RPMI medium and treated with 100 μM final concentration of hydrogen peroxide for 0, 1, 2 and 4 hours. Cells were harvested and stored at -80°C until used to purify Tc DNA polymerase β, western blot analysis, activity gels and mRNA purification.

### Epimastigote cell culture and H_2_O_2_ treatment

Epimastigote cells were grown to log phase in LIT media as described [49]. The cells were centrifuged and resuspended at 2 x 10^8^ epimastigotes/ml in fresh media with 10% fetal bovine serum. Hydrogen peroxide was added at a final concentration of 100 μM with incubation for 0, 1, 2 or 4 hours at 28°C. After incubation, the cells were harvested and stored at -80°C until used to obtain cell extracts for activity gels, western blot analysis, Tc polymerase β purification and mRNA purification.

### Western blot analysis

Samples containing epimastigote or trypomastigote cells were resuspended in 20 μl of 20 mM Tris-HCl (pH 7.5), 0.5 mM EDTA, 0.1% v/v Triton X-100, 0.1% v/v NP-40, 200 mM NaCl and 0.5 mM PMSF. The cells were broken by mild sonication and centrifuged at 12000 rpm for 15 minutes. The supernatant was collected and mixed with Laemli sample buffer and heated at 100°C for 5 minutes. Proteins were separated in 8.5% SDS-PAGE gels and transferred to PVDF membranes (Immobilon, USA); *T*. *cruzi* DNA β polymerase was detected using anti-DNA β polymerase or anti-phospho aminoacid (Abcam, UK). Detection of the antibodies was performed by anti-rabbit IgG conjugated to alkaline phosphatase (Promega, USA) followed by chemiluminescence (Novex, USA) or colorimetric assay (BioRad, USA).

### DNA polymerase activity gel

Detection of DNA synthesis on SDS-PAGE was done according to the literature [50], with slight modification. Briefly, protein samples from 1 x 10^6^ epimastigote and 2,5 x 10^6^ trypomastigote cells treated or not with 100 μM H_2_O_2_, were mixed with fetal calf serum (10% v/v final concentration) and 0,2 volumes of Laemmli sample buffer containing 10 mM DTT. Then, samples were heated at 37°C for 5 minutes. Proteins were separated on an 8.5% SDS-PAGE gel containing 100 μg/ml activated calf thymus DNA, and after the run was complete, the gels were washed twice with 50 mM Tris-HCl (pH 8.0) at room temperature. Then the proteins in the gel were renatured at room temperature for 3 hours in a folding buffer (50 mM Tris–HCl pH 8.0, 0.5 mM EDTA, 5 mM DTT, 0.5 mg/ml BSA, 15% v/v glycerol, 0.01% v/v NP-40 and 4 mM Mg-Acetate). Then the gel was incubated at 4° overnight with folding buffer to renature the proteins further. After this step the gel was incubated overnight in folding buffer supplemented with 250 mM KCl, 25 μM dTTP, dGTP, dCTP, 1 μM dATP, 1 μl/ml P^32^ dATP (250 μCi/mMol) and 3 mM MnCl_2_. The incubation was done at room temperature with gentle agitation. Then the gel was washed at least five times with 5% v/v TCA and 2% w/v potassium pyrophosphate for 30 minutes at room temperature each wash. A final wash was done with the above solution at 50°C for 45 minutes and the gels were exposed to X-ray film for autoradiography. Those SDS-PAGE activity gels used to analyze the H_2_O_2_ response were supplemented with 4 mM MnCl_2_ in order to obtain a better activity of the enzyme.

### Chromatin inmunoprecipitation and end point PCR analysis

Epimastigote (63 x 10^6^ cells) and trypomastigote (5 x 10^6^ cells) incubated by 4 hours with 100 μM H_2_O_2_ were washed with sterile PBS, resuspended in 10 ml PBS and treated with 200 μl formaldehyde (37% w/v) for 4 minutes at room temperature. Crosslinking of the proteins to the DNA was quenched by adding 1 ml 1 M glycine. The cells were centrifuged and washed with PBS. After washing, cells were resuspended in 600 μl RIPA buffer (1% v/v NP-40, 0.5% w/v deoxycholate, 0.1% w/v SDS, 150 mM NaCl, 1 mM EDTA, 1 mM EGTA and 50 mM Tris-HCl pH 8.0) and the mix was left to stand for 15 minutes at room temperature. The mix was sonicated to produce DNA fragments of approximately 1 Kb average length. An aliquot of 100 μl was kept to purify the input DNA. Another aliquot of 500 μl was precleared with 40 μl of protein A–agarose (Invitrogen, USA) beads previously washed in RIPA buffer. The mix was incubated for 3 hours at 4°C. After centrifuging the extract, the supernatant was incubated with 2 μg of anti-Tc DNA polymerase β antibodies and 40 μl Protein A–agarose beads previously washed in RIPA buffer. Incubation was performed at 4°C for 8 hours. Then the mix was centrifuged and the beads were washed 4 times with 50 mM Tris-HCl (pH 8.0) containing 1 mM EDTA, 150 mM NaCl and 0.1% v/v Triton X-100. A final wash was made with PBS. The beads were resuspended in 200 μl 60 mM Tris-HCl (pH 6.8), 200 mM NaCl, 2% w/v SDS and 10 mM DTT. The beads were finally incubated for 8 hours at 65°C to reverse the crosslink. Supernatants containing DNA were extracted once with phenol-chloroform and once with chloroform to eliminate proteins. Precipitated DNA was obtained by adding 0.1 volume Na-acetate pH 5.2 and 3 volumes ethanol and after centrifuging the pellet was washed with 80% ethanol, dried and resuspended in 10 μl TE buffer. The DNA was amplified by PCR using specific primers as indicated in Table 1.

### Quantitative reverse transcriptase Real-Time PCR

Total RNA was isolated from 63 x 10^6^ epimastigote cells or 5 x 10^6^ trypomastigote cells previously treated with hydrogen peroxide for 0, 2 or 4 hours. Cells were resuspended in 1 ml of TRizol Reagent (Ambion, USA) and the RNA was isolated as indicated by the manufacturer. The precipitated RNA was resuspended in 20 μl of TE buffer and digested with 1 unit of DNAase free of RNAase (Promega, USA). The DNAase was inactivated at 70°C for 20 minutes. Reverse transcription was performed using 1 μg of RNA as template and following manufacturer’s instruction (NEB, USA). Tubuline (internal control) and *T*. *cruzii* DNA polymerase β were amplified using equal amounts (50 ng) of cDNA from each treatment as template and using Brillant II Sybr Green GPR Master Mix (Agilent, USA). PCR reactions were performed in the following conditions: initial denaturation of 10 minutes at 95°C, 35 cycles of 10 seconds at 95°C, 10 seconds at 53°C and 30 seconds at 72°C, with a final elongation at 72°C for 5 minutes. The primers used are described in Table 1.

### Northern blot

The integrity of 2 μg of RNA of each sample was analyzed on a 1% agarose-formaldehyde gel. RNA samples were then transferred to a Hybond membrane according to manufacturer’s instructions (GE Healthcare Life Sciences, USA). Nucleic acids were crosslinked to membranes using UV light (UV Stratalinker 2400, Stratagene, USA). Membranes containing RNA samples were washed for 10 minutes with 4X SSC buffer (600 mM NaCl, 60 mM sodium citrate pH = 7.2) and then incubated for 2 hours at 48°C with pre-hybridization buffer (750 mM NaCl, 75 mM sodium citrate, 50% formamide, 0.2% SDS, 200 μg/ml salmon sperm DNA, 5X Denhart’s solution [0.1% Bovine serum albumin, 0.1% ficoll 400, 0.1% polyvinylpyrrolidone], pH = 7.2). Then membranes were incubated overnight at 48°C with probes to detect Tc DNA pol β or actin previously labeled with dUTP-digoxigenin according to manufacturer’s instructions (Roche, Germany) and denatured at 110°C for 10 minutes and mixed with ultrahyb (Thermofisher, USA) at 50 ng/ml each probe. Primers used to generate probes are listed in Table 1. Membranes were washed twice for 5 minutes at 48°C, first with 2X SSC/SDS (300 mM NaCl, 30 mM sodium citrate, 0.4% SDS, pH = 7.2) and then with 0.5X SSC/SDS (75 mM NaCl, 0.75 mM sodium citrate, 0.4% SDS, pH = 7.2). Then membranes were washed twice at room temperature for 5 minutes with maleic acid buffer supplemented with Tween-20 (50 mM maleic acid, 150 mM NaCl, 0.3% Tween-20, pH = 7.5). Membranes were blocked with 1% non-fat milk in maleic acid buffer for 1 hour at room temperature. Then, membranes were incubated with anti-digoxigenin alkaline phosphatase (AP) conjugated (Roche, Germany; diluted 1/10000 in maleic buffer alone) for 1 hour at room temperature. After this, membranes were washed twice for 5 minutes with maleic acid buffer supplemented with Tween-20. Then after incubation for 2 minutes with AP buffer (100 mM Tris pH = 9.5, 100 mM NaCl, 5 mM MgCl_2_), membranes were incubated with AP substrate for chemiluminisence (Novex, USA) for 5 minutes. Membranes were exposed on an X-ray film (Santa Cruz Biotechnology, USA) for different times to evaluate signals, which were quantified using Image J software (NIH, USA).

### Affinity purification of Tc DNA beta polymerase from cell extracts by affinity chromatography

Purified antibodies against Tc DNA polymerase β/27] were crosslinked in a 1 mg/ml settled protein A-agarose using dimethyl pimelimidate (Pierce, USA). After crosslinking the resin was treated with 0.1 M glycine-HCl (pH 2.5) and quickly neutralized with 0.5 M Tris-HCl (pH 8.0) followed by incubation in equilibration buffer (50 mM Tris-HCl (pH 8.0), 500 mM KCl, 0.5 M EDTA, 0.5 mM EGTA, 1 mM DTT, 0.1% v/v NP-40 and 10% v/v glycerol). Epimastigote cell extract (2 ml) containing 4 mg of protein was incubated with 0.4 ml of the affinity resin at 4°C for 8 hours. Then the resin was packed into a disposable Bio-Rad column and extensively washed with equilibration buffer. A final wash was given using 8 ml of TE buffer supplemented with 0.5 mM DTT. Bound Tc DNA polymerase β was eluted using a buffer containing 25 mM Tris–HCl (pH 6.8), 3 M urea, 1% w/v SDS and 1 mM DTT. Fractions of 300 μl were collected and concentrated to 50 μl using a Speed-Vac (Savant, USA). These fractions were analyzed by Western blot or used in activity gels.

### Statistical analysis

Differences between means in all data presented in this work were analyzed for statistical significance using Student’s t-tests in the Prism software (GraphPad Software, USA). Significance was considered when *p* < 0,05.

## Supporting information

S1 Data This supporting file is showing the complete dataset of all the experiments performed in this work and showed in graphics present in Figs 1B, 2B, 3D and 4. (XLSX)
